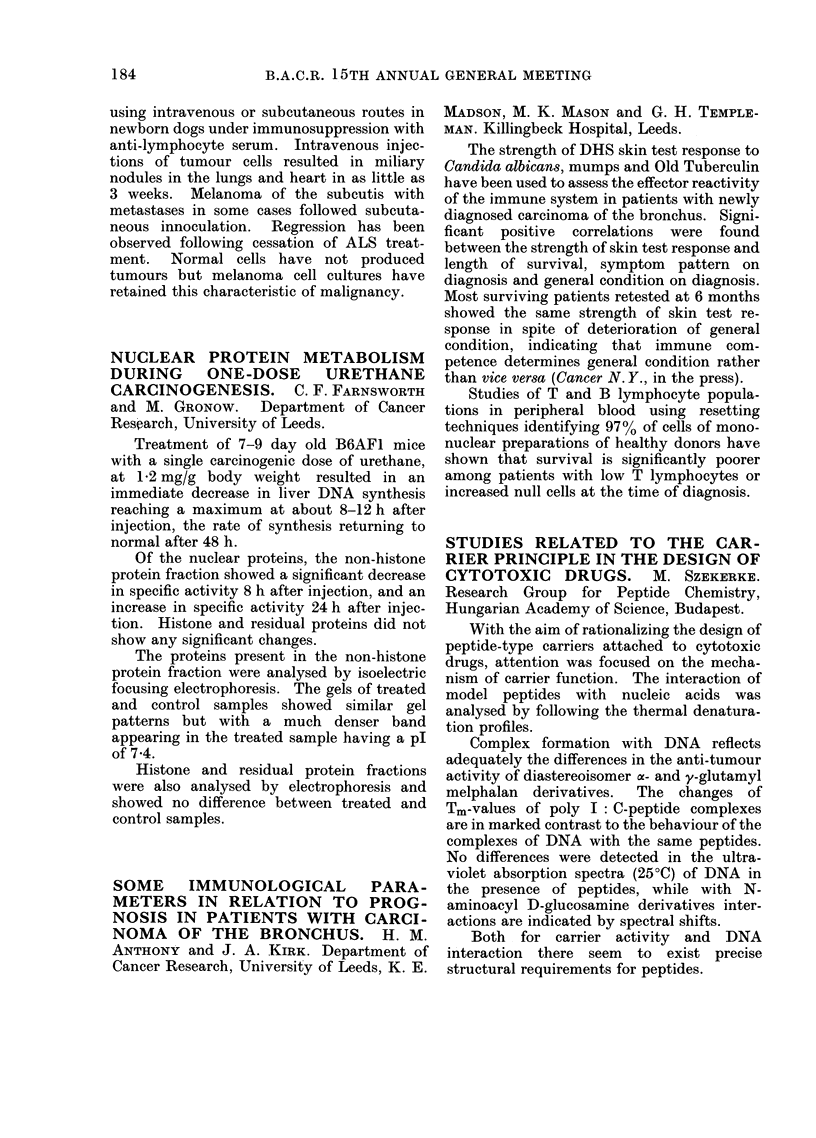# Proceedings: Nuclear protein metabolism during one-dose urethane carcinogenesis.

**DOI:** 10.1038/bjc.1974.169

**Published:** 1974-08

**Authors:** C. F. Farnsworth, M. Gronow


### NUCLEAR PROTEIN METABOLISM DURING ONE-DOSE URETHANE CARCINOGENESIS.

C. F. Farnsworth and M. Gronow. Department of Cancer Research, University of Leeds.

Treatment of 7–9 day old B6AF1 mice with a single carcinogenic dose of urethane, at 1·2 mg/g body weight resulted in an immediate decrease in liver DNA synthesis reaching a maximum at about 8–12 h after injection, the rate of synthesis returning to normal after 48 h.

Of the nuclear proteins, the non-histone protein fraction showed a significant decrease in specific activity 8 h after injection, and an increase in specific activity 24 h after injection. Histone and residual proteins did not show any significant changes.

The proteins present in the non-histone protein fraction were analysed by isoelectric focusing electrophoresis. The gels of treated and control samples showed similar gel patterns but with a much denser band appearing in the treated sample having a pI of 7·4.

Histone and residual protein fractions were also analysed by electrophoresis and showed no difference between treated and control samples.